# Automatic Fabric Defect Detection with a Multi-Scale Convolutional Denoising Autoencoder Network Model

**DOI:** 10.3390/s18041064

**Published:** 2018-04-02

**Authors:** Shuang Mei, Yudan Wang, Guojun Wen

**Affiliations:** School of Mechanical Engineering and Electronic Information, China University of Geosciences, Wuhan 430074, China; meishuang@hust.edu.cn (S.M.); wangyodan@163.com (Y.W.)

**Keywords:** fabric defect detection, unsupervised learning, deep neural network, convolutional denoising autoencoder, Gaussian pyramid

## Abstract

Fabric defect detection is a necessary and essential step of quality control in the textile manufacturing industry. Traditional fabric inspections are usually performed by manual visual methods, which are low in efficiency and poor in precision for long-term industrial applications. In this paper, we propose an unsupervised learning-based automated approach to detect and localize fabric defects without any manual intervention. This approach is used to reconstruct image patches with a convolutional denoising autoencoder network at multiple Gaussian pyramid levels and to synthesize detection results from the corresponding resolution channels. The reconstruction residual of each image patch is used as the indicator for direct pixel-wise prediction. By segmenting and synthesizing the reconstruction residual map at each resolution level, the final inspection result can be generated. This newly developed method has several prominent advantages for fabric defect detection. First, it can be trained with only a small amount of defect-free samples. This is especially important for situations in which collecting large amounts of defective samples is difficult and impracticable. Second, owing to the multi-modal integration strategy, it is relatively more robust and accurate compared to general inspection methods (the results at each resolution level can be viewed as a modality). Third, according to our results, it can address multiple types of textile fabrics, from simple to more complex. Experimental results demonstrate that the proposed model is robust and yields good overall performance with high precision and acceptable recall rates.

## 1. Introduction

**Figure 1:**
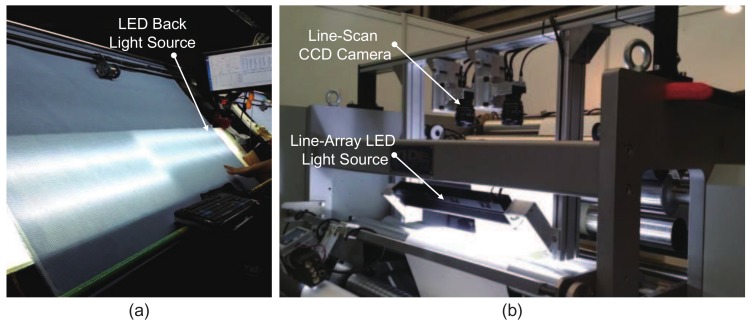
Fabric defect detection by (**a**) manual and (**b**) [4] automated optical inspection methods.

A fabric is a textile material, short for “textile fabric” [1], that is manufactured with textile fibers and widely used in daily life. A fabric defect is a flaw on the fabric surface resulting from the manufacturing process [2]. Unlike other processes, quality inspection of the fabric surface is highly important to textile manufacturers before products reach customers. Traditionally, visual inspection performed by experienced human inspectors has been used to ensure fabric quality, as shown in Figure 1a. Limited by factors such as human fatigue and inattentiveness, visual detection methods can hardly provide reliable and stable results [3]. Moreover, these results are usually subjective and cannot be quantified.

In recent years, with the rapid development of machine vision and digital image processing techniques, automated fabric defect inspection has become popular and has gradually displaced the traditional manual method. As shown in Figure 1b, automated inspection of fabric defects usually involves three steps: image acquisition, defect detection and post-processing. The image acquisition procedure is mainly responsible for the digital image capture of defective samples, and generally, a line-scan charge coupled device (CCD) camera can be used. The defect detection procedure is performed to localize and segment the flawed regions, and sometimes, it also includes quantitative analysis. The last procedure refers to all subsequent processes after defect detection, e.g., defect type classification and defect grade assessment. In this paper, we mainly concentrate on the defect detection step, which is considered more challenging in fabric quality inspection.

**Figure 2:**
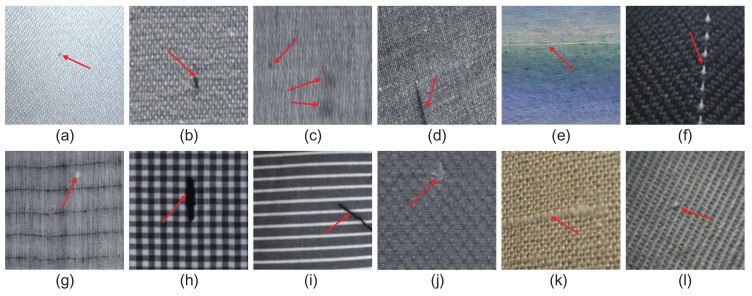
Defective fabric samples with different patterned textures.

**Figure 3:**
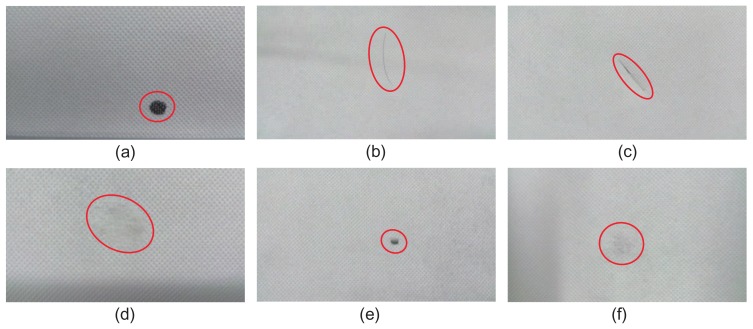
Different types of defects in cotton fabric; from (**a**) to (**f**), the defects are classified as oil polluted, heterozygous, scratched, flying-fiber, perforated and gauzy types.

Three main challenges exist in the defect detection task. First, there are a broad range of different fabrics, which usually exhibit varied characteristics. As making general algorithms compatible with various texture types is difficult, instability in the traditional fabric defect detection methods may occur. According to [2,5], all fabrics can be classified using up to 17 established wallpaper groups dominated by *p*1, *p*2, *p*3, p3m1, p31m, p4, p4m, p4g, pm, pg, pmg, pgg, p6, p6m, cm, *cmm* and *pmm*, which have lattices based on parallelogram, rectangular, rhombic, square, or hexagonal shapes. In Figure 2, we show some common defective fabric samples with different 2-D patterned textures. All this variability increases the complexity of the defect detection problem, making it difficult to devise a generalized method. Second, the categories and characteristics of fabric defects themselves are generally varied. Currently, more than 70 categories of fabric defects defined by the textile industry exist [2]. These defects can be caused by different factors, such as machine malfunctions, yarn problems, and oil stains [6]. As shown in Figure 3, these defects can have vastly different manifestations in the same category of fabric (the p1 group). Some defects, e.g., the ones in Figure 3a,c,d,f, appear as regions of low contrast, nonuniform brightness, or irregular shape, which further contributes to the difficulties. Third, collecting large numbers of fabric defect samples, especially some rare types, is extremely difficult in industry, resulting in a data imbalance or a complete failure for some traditional supervised methods.

Though challenging, numerous researchers have devoted substantial efforts to these issues. Considering the different appearances of inspected fabrics, Ngan et al. [2] broadly categorized the methods into two main groups, non-motif-based and motif-based methods. Most works focus on the non-motif-based group [7,8,9,10,11,12]. Specifically, Bissi et al. [13] presented an algorithm for automated texture defect detection in uniform and structured fabrics based on a complex symmetric Gabor filter bank and principal component analysis (PCA). Experimental results using the TILDA textile texture database have verified the robustness and computation-saving performance of this method. However, this method does not generalize well for some slightly complex patterned textures. Harinath et al. [14] proposed a wavelet transform-based method for fabric defect detection, which is well suited for quality inspection due to its multi-resolution feature extraction. However, similar spectral approaches are usually computationally demanding. Qu et al. [15] proposed a defect detection algorithm for fabrics with complex textures based on a dual-scale over-complete dictionary. This method can enhance the self-adaptability of defect detection by considering large variations in the defect sizes. It also achieved excellent detection performance on comparison datasets. However, this method requires the inspected images to be aligned with the training samples in the dictionary. In addition, it is not efficient in detecting low contrast defects. In addition to these non-motif-based methods, only a few studies have conducted the defect detection task by considering elementary fabric motifs as a basic manipulation unit (motif-based) [2]. These methods usually require a defect-free ground truth for comparison of the motifs, or they analyze the energy of motif subtraction to highlight defects [16]. This method is not robust and can be time consuming. In addition, it is generally not suitable for the *p*1 group (this group refer to fabric which is composed of one fundamental lattice with one motif only). Therefore, we will mainly concentrate on the non-motif-based models in later discussions and experiments.

In this paper, we present a novel unsupervised learning-based model that is capable of coping with different types of defects in the *p*1 and non-*p*1 groups. This model is a multi-scale convolutional denoising autoencoder (MSCDAE) architecture. The inputs into the network at each scale are generated by a Gaussian pyramid to cope with defects of different sizes. Instead of considering elementary motifs as a basic manipulation unit, this model tries to train the multiple convolutional denoising autoencoder (CDAE) networks with randomly sampled image blocks (also known as image patches) from defect-free samples. In fabric samples of the same type, these image patches, which do not contain defective areas, are usually highly similar. Therefore, after training, the CDAE network is capable of modeling the distribution of these defect-free image patches in the patch domain. Filters in the trained CDAE network will be sensitive to similar patches and thus show large responses to them. For patches that contain defective areas, their appearances and distributions in the patch domain will usually be quite different. The trained model may therefore be less sensitive to them, and relatively small responses will be generated. By measuring the residual between the response and the original input, direct pixel-wise prediction can easily be conducted. Finally, by synthesizing prediction results from multiple pyramid layers, the final inspection representation for a candidate sample can be generated.

The main contributions of this paper are summarized in the following points.

- We proposed a new non-motif-based method MSCDAE which has the advantage of good compatibility for fabric defect detection. This method is a learning-based model that is suitable for the *p*1 and non-*p*1 types of fabrics. Experimental results have verified its good performance.
- The multi-pyramid and CDAE architectures in this model are novel and subtle. Specifically, processing in a multi-scale manner with pyramids may ensure the capture of sufficient textural properties, which are often data independent. In addition, applying the CDAE network can distinguish defective and defect-free patches easily through the use of reconstruction residual maps, which are more intuitive.
- This model is conducted in an unsupervised way, and no labeled ground truth or human intervention is needed. Furthermore, only defect-free samples are required for the training of this model. All these properties make it easier to apply the method in practice.

The remainder of this paper is organized as follows. In Section 2, we briefly review the fabric defect detection methods and the foundations of the CDAE network. The reconstruction residual of this network serves as the core indicator of direct pixel-wise prediction for defect detection in later experiments. Then, in Section 3, procedures of the proposed MSCDAE model are described in detail, and steps to train this model and test candidate images are summarized. In Section 4, the overall performance of the proposed method is analyzed and compared with other well-known defect inspection methods. Relevant points about parameter selection are also discussed in this section. Implementation details of the proposed method are illustrated in Section 5. Finally, we give our conclusions in Section 6.

## 2. Related Works and Foundations

**Figure 4:**
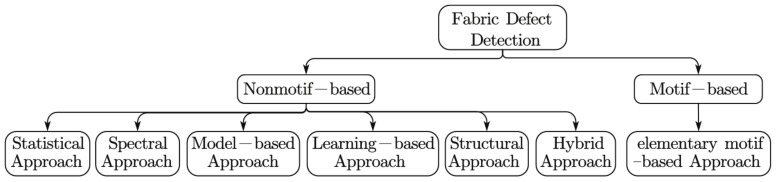
Different approaches for patterned fabric defect detection.

As previously stated, traditional fabric defect detection methods can be categorized into two main groups: non-motif-based and motif-based groups. According to [2,6,8,11], the majority of these methods belong to the first group. In this group, methods can be further subdivided into six categories: statistical, spectral, model-based, learning-based, structural and hybrid approaches [2], as shown in Figure 4. The statistical approach is a widely used method that seeks to distinguish defective and defect-free regions according to their different statistical characteristics, e.g., similarity, uniformity, and regularity. Typical statistical approaches include the auto-correlation metric, co-occurrence matrix and fractal dimension methods. The spectral approach is another widely used method for defect inspection that highlights the difference between defective and defect-free regions in the frequency domain. Fourier transformation, wavelet transformation and Gabor transformation are all typical spectral methods [17,18]. Though effective, these methods are usually computationally demanding. Model-based and structural approaches are relatively less common, possibly because these two approaches are highly data dependent. The models designed or the texture primitives utilized are varied for different types of patterned textures. The hybrid approach is a combined architecture that integrates two or more strategies for defect inspection [11,13,19]. Hybrid approaches are usually capable of coping with different types of defects, and their inspection performance tends to be more effective and robust. However, these methods also have high time and computation costs. Moreover, it is generally difficult to design such a generalized approach. Motif-based approaches are methods that consider elementary motifs as basic manipulation units. These methods generally have better generality than the non-motif-based methods. However, currently, they cannot tackle the patterned texture of the *p*1 group, e.g., plain and twill fabrics, very well. Additionally, they tend to be sensitive to noise and nonuniform illumination in situations in which the working environment is relatively poor. Here, we will mainly concentrate on the learning-based approach.

Learning-based approaches, especially methods with deep neural network architectures, are very promising for defect inspection. In recent years, there have been many studies that have investigated this field and explored better strategies for defect inspection [20,21,22,23]. However, the majority of these studies use supervised learning, which often requires large amounts of labeled defective samples for model training [23]. The autoencoder (AE) network is a typical unsupervised method that has been widely used in shape retrieval [24], scene description [25], target recognition [26,27] and object detection [28]. It can be trained without any labeled ground truth or human intervention. Since it is also an important component of the proposed model, the foundations and developments of this network will be briefly described below.

AE networks are based on an encoder-decoder paradigm that is usually trained in an unsupervised fashion and in a fully connected form. Convolutional autoencoder (CAE) networks differ from conventional AE networks in that they retain the structure information of the 2-D images and the weights are shared among local positions. The architecture of a typical CAE contains an encoder part with convolutional and pooling layers and an analogous decoder part with deconvolutional and upsampling layers. The encoder and decoder parts can be defined as transitions ϕ and ψ such that:

(1) $$\begin{array}{c} \phi :X\to F,\\ \psi :F\to X,\\ \phi ,\;\psi =\arg\min_{\phi ,\;\psi }{∥x-\psi \left(\phi \left(x\right)\right)∥}^{2}, \end{array}$$

where $x\in R^{d}=X$ refers to an image patch in the X domain, and $z=\phi \left(x\right)\in R^{p}=F$ refers to the corresponding hidden layer map in the F domain. Assume that $x^{\prime }$ denotes the reconstruction; then, the encoder and decoder processes can be expanded as:

(2) $$\begin{array}{cc} z= & \sigma \left(W\circ x+b\right),\\ x^{\prime }= & \sigma^{\prime }\left(W^{\prime }\circ z+b^{\prime }\right), \end{array}$$

where “∘” is the convolution process, W and $W^{\prime }$ are the weight matrices, b and $b^{\prime }$ are the bias vectors for the encoder and decoder, respectively, and σ and $\sigma^{\prime }$ are the nonlinear mapping processes, specifically, the convolutional, pooling, deconvolutional, and upsampling processes. Particularly, the pooling and upsampling processes are usually conducted in the form of max-pooling and max-unpooling [29]. The CAE model can be trained to minimize the reconstruction errors (such as the mean squared errors):

(3) $$\begin{array}{c} L\left(x,x^{\prime }\right)=\frac{1}{2N}\sum_{i=1}^{N}{∥x_{i}-x_{i}^{\prime }∥}^{2}+\lambda \cdot \sum_{w\in \left\{{W,W}^{\prime }\right\}}{∥w∥}_{F}, \end{array}$$

where *N* is the number of samples, λ is a constant that balances the relative contributions of the reconstruction and the regularization terms, and $\sqrt{{∥x_{i}-x_{i}^{\prime }∥}^{2}}$ is the reconstruction residual of the *i*-th image patch.

**Figure 5:**
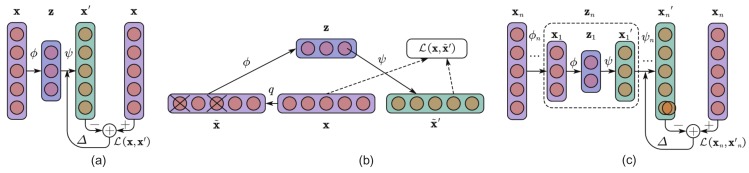
Architectures of the (**a**) AE; (**b**) denoising AE; and (**c**) stacked AE models.

The CDAE network is slightly different from the CAE in that it takes partially corrupted inputs for model training and aims to recover the original undistorted inputs. This is done by first corrupting the initial input x into $\tilde{x}$ by means of a stochastic mapping $\tilde{x}∼q\left(\tilde{x}|x\right)$. Assume that ${\tilde{x}}^{\prime }$ is the reconstruction of the corrupted data $\tilde{x}$; then, loss in the CDAE model is measured by the reconstruction error $L\left(x,{\tilde{x}}^{\prime }\right)$, as shown in Figure 5b. The concrete form of $L\left(x,{\tilde{x}}^{\prime }\right)$ is similar to that of $L\left(x,x^{\prime }\right)$ in Equation (3). In general, the CDAE model is conducted in a stacked form, which allows hierarchical feature extraction from unlabeled samples, as shown in Figure 5c. A stochastic gradient descent algorithm [30] can be easily used to optimize of all these neural network models.

## 3. Proposed Methods

**Figure 6:**
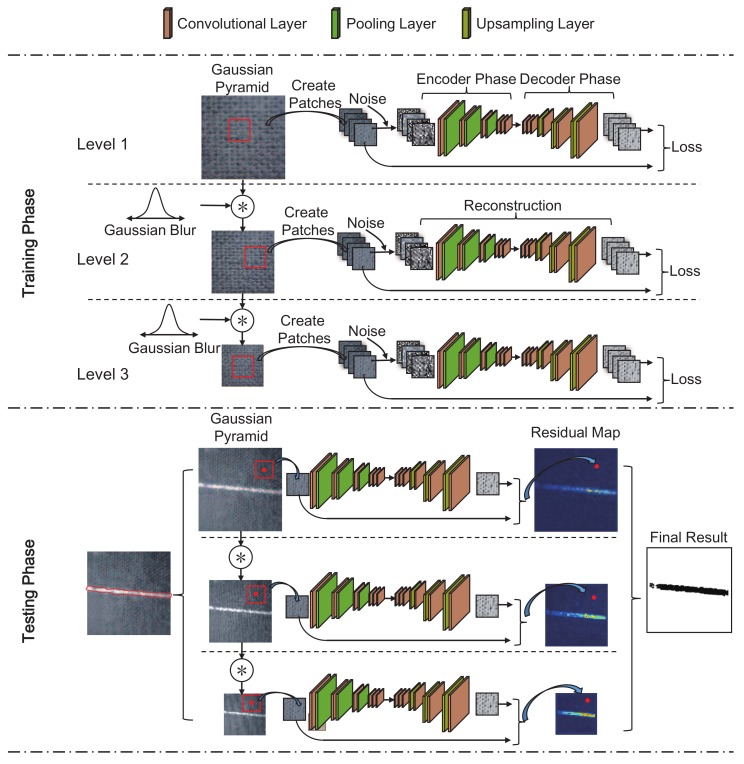
Overall architecture of the proposed MSCDAE model.

In this section, procedures of the proposed MSCDAE model are discussed in detail. Figure 6 shows the overall architecture of this model in the training and testing phases. Procedures in the training phase mainly aim to learn the CDAE network in each pyramid level and calculate the optimal threshold for defect segmentation, while those in the testing phase are processes used to inspect a candidate defective image. Specific illustrations are presented as follows.

### 3.1. MSCDAE Model Training

The training phase of the CDAE network in each pyramid level is conducted separately in the proposed MSCDAE model. Specifically, this training phase mainly includes the image preprocessing, patch extraction, model training and threshold determination procedures.

#### 3.1.1. Image Preprocessing

All image preparations prior to the training procedure are included in this part: illumination normalization, Gaussian pyramid downsampling, and noise corruption steps.

*Illumination normalization*: In general, in most existing methods, the defect inspection process is highly sensitive to illumination variations. To reduce the rate of false detection, an illumination normalization process based on Weber’s law [31] is first conducted. According to this law, stimuli are perceived not in absolute terms but in relative terms. Assume that I is an image to be inspected; then, the illumination normalization process can be implemented as:

(4) $$I^{\prime }=WLD\left(I\right),$$

where WLD(·) refers to a Weber local descriptor [32], which can be expressed as:

(5) $$WLD\left(I\right)=\arctan\left(\sum_{\Delta x\in A}\sum_{\Delta y\in A}\frac{I\left(x,y\right)-I\left(x-\Delta x,y-\Delta y\right)}{I\left(x,y\right)}\right),$$

where A={-1,0,1}, and {(x-Δx,y-Δy)|Δx∈A,Δy∈A} refers to the 8-neighborhood region of point (x,y) (note that this process is redundant in the case where the changes in brightness are due to a defect).

*Gaussian pyramid downsampling*: In a Gaussian pyramid, subsequent images are weighted using a Gaussian blur and scaled down by resampling, as shown in Figure 6. Each pixel in a pyramid level contains a local average that corresponds to a pixel neighborhood in a lower level of the pyramid. That is:

(6) $$I^{\left(n+1\right)}=S^{↓}G\left(I^{\left(n\right)}\right),\;n=1,2,\cdots n_{l};\;I^{\left(1\right)}=I^{\prime },$$

where $S^{↓}$ refers to the downsampling process, G(·) denotes the Gaussian convolution, $n_{l}$ is the number of pyramid levels, and $I^{\prime }$ is the image after illumination normalization.

*Noise corruption*: The salt and pepper noise model is utilized for data corruption in the MSCDAE model. Let ${\tilde{I}}^{\left(n\right)}$ denote the corrupted image at level *n* and ${\tilde{g}}_{i,j}$ and $g_{i,j}$ refer to the gray level of the pixel at position (i,j) in the corrupted and original clean images. The corrupted data are given by:

(7) $${\tilde{g}}_{i,j}=\left\{\begin{array}{cc} g_{i,j}, & \mathrm{with}\;\;\mathrm{probability}\;\;\left(1-p\right)\\ s, & \mathrm{with}\;\;\mathrm{probability}\;\;p\;\;\;\;\;\;\;\;\;\;\;\;\;\; \end{array},\right.$$

where

(8) $$s=\left\{\begin{array}{cc} 0, & \mathrm{with}\;\;\mathrm{probability}\;\;0.5\\ 255, & \mathrm{with}\;\;\mathrm{probability}\;\;0.5 \end{array}.\right.$$

The probability *p* directly affects the degree of data contamination, and it can be determined by cross-validation [33].

#### 3.1.2. Patch Extraction

Patch extraction in this phase refers to the process used to create training sets for the proposed model. For pixel-wise prediction, characterizing a pixel based on local neighborhood information can be more robust than using only a single pixel. Nevertheless, the size of the local neighborhoods is usually data dependent. Owing to the multi-scale pyramid structure, fixed size patch extraction can be conducted at multi-scale levels instead of generating patches with various sizes, which is computationally expensive. In this phase of model training, patches can be collected randomly from the training samples. However, it should be noted that only defect-free images are utilized for model training and that the patch set at each pyramid layer should not be confused with those from other layers. After preparing training sets for each corresponding CDAE network, the model training process can be conducted.

#### 3.1.3. Model Training

As previously stated, patches that come from either defect-free or defective regions elicit very different responses in the CDAE network after training. Therefore, it is natural to use the reconstruction residual as the criterion for pixel-wise prediction in defect detection. Training of the proposed MSCDAE network aims to model the distribution of defect-free image patches by minimizing the total reconstruction loss of all patches in each image pyramid layer. In this paper, the optimization process is conducted by applying a batch gradient descent algorithm in an error back propagation fashion. We illustrate the procedures of one iteration epoch with *m* batches as follows:
   *Step 1*: ∀l, set $△W^{\left(l\right)}=0,△b^{\left(l\right)}=0;$   *Step 2*: for i=1 to *m*,       *a.* Calculate the partial derivatives $▽{\;}_{W^{\left(l\right)}}L\;\left(x,x^{\prime }\right)$ and $▽{\;}_{b^{\left(l\right)}}L\left(x,x^{\prime }\right);$       *b.* Partial differential superposition:          $△\;W^{\left(l\right)}=△\;W^{\left(l\right)}+▽{\;}_{W^{\left(l\right)}}L\left(x,x^{\prime }\right),$
$△b^{\left(l\right)}=△b^{\left(l\right)}+▽{\;}_{b^{\left(l\right)}}L\left(x,x^{\prime }\right);$   *Step 3*: Update weight parameters:       *a.* Renew $W^{\left(l\right)}=\;W^{\left(l\right)}-\alpha \cdot △\;W^{\left(l\right)}$, $b^{\left(l\right)}=\;b^{\left(l\right)}-$
$\alpha \cdot △\;b^{\left(l\right)}$;       *b.* Disrupt the order of patches in the dataset and finish the current iteration epoch;
where *l* denotes the *l*-th layer of the deep network, and α is the learning rate, which will be discussed in later experiments. Procedures to optimize the parameters $W^{\prime }$ and $b^{\prime }$ are similar and will not be discussed here.

#### 3.1.4. Threshold Determination

The segmentation threshold is a parameter that measures the reconstruction error and determines if the corresponding image patch is defect-free or defective. The goal of threshold determination is to find the optimal threshold value that achieves the trade-off between reducing the rate of false negatives (points that are defective but judged to be defect-free) and reducing the rate of false positives (points that are defect-free but judged to be defective) simultaneously. In the proposed MSCDAE model, the segmentation threshold at each pyramid level is determined according to the corresponding training set. Assume that $\xi^{\left(i\right)}\;\;=\;\;\left\{\vartheta^{\left(i\right)}\left(x_{k}\right)|k\;=\;1,2,\cdots ,N_{p}\right\}$ refers to the reconstruction residual for a set of patches in the *i*-th image pyramid layer, $N_{p}$ gives the total number of training patches, $x_{k}$ is the *k*-th image patch, and $\vartheta^{\left(i\right)}\left(x_{k}\right)=\sqrt{{∥x_{k}-x_{k}^{\prime }∥}^{2}}$ is the reconstruction residual between $x_{k}$ and $x_{k}^{\prime }$. Note that the $\vartheta^{\left(i\right)}$ values for the majority of defect-free pixels will be mainly concentrated near the mean value and their distribution will be close to the Gaussian distribution. Therefore, the segmentation threshold can be defined as $T^{\left(i\right)}=\mu^{\left(i\right)}+\gamma \cdot \sigma^{\left(i\right)}$, where $\mu^{\left(i\right)}$ and $\sigma^{\left(i\right)}$ are the mean and standard deviation of set $\xi^{\left(i\right)}$. The parameter γ can be adjusted according to the segmentation sensitivity. In later experiments, we will set γ=2 according to experimental verification. Note that the thresholds are determined by using the training patches, which are all defect-free.

After model training, novelty detection and defect localization processes can be easily conducted for a candidate textural image. Detailed procedures are presented below.

### 3.2. MSCDAE Model Testing

The testing phase of the MSCDAE model is shown in Figure 6. This phase mainly includes the image preprocessing, patch extraction, residual map construction, defect segmentation and synthesization procedures.

#### 3.2.1. Image Preprocessing

The steps in the image preprocessing parts of the testing phase are slightly different from those in the training phase in that the noise corruption step is not necessary for inspecting a candidate image. As for the illumination normalization and Gaussian pyramid downsampling steps, the implementations are the same.

#### 3.2.2. Patch Extraction

After preprocessing, patches will be extracted such that the textural images can be inspected. In the testing phase, this process should be strictly conducted row by row or column by column to maximize convenience when generating the residual map [34] (in the training phase, patches can be extracted randomly). Suppose the patch size is w×h and the stride interval is *s*. The dimension of the patch set generated from an image with size W×H can be expressed as $\left[N_{p},N_{c},w,h\right]$, where $N_{p}=\left\lceil \left[\left(W-w\right)/s+1\right]\right\rceil \times \left\lceil \left[\left(H-h\right)/s+1\right]\right\rceil$ refers to the number of patch samples and $N_{c}\in \left\{1,\;3\right\}$ refers to the number of image channels. The extraction strategy and parameters (w,h and *s*) applied are the same as those in the training phase.

#### 3.2.3. Residual Map Construction

To predict a pixel, a local receptive field, x, with size w×h in its neighborhood will be extracted and then passed forward to the trained model. This is shown in Figure 6. The reconstruction residual $\vartheta \left(x\right)$ serves as the criterion and represents the extent to which the point belongs to a defective area. Therefore, in each image pyramid layer, a residual map can be constructed for subsequent processing.

#### 3.2.4. Defect Segmentation

Defect segmentation is conducted on the residual map in each image pyramid layer as follows:

(9) $$\varsigma^{\left(i\right)}\left(x_{j,k}\right)=\left\{\begin{array}{c} 0\\ 1 \end{array}\;\;\begin{array}{c} \mathrm{if}\;\vartheta^{\left(i\right)}\left(x_{j,k}\right)\leq T^{\left(i\right)}\\ \mathrm{otherwise} \end{array},\right.$$

where $\vartheta^{\left(i\right)}\left(x_{j,k}\right)$ refers to the reconstruction residual at position (j,k) in the *i*-th layer, and $\varsigma^{\left(i\right)}\left(x_{j,k}\right)$ is the corresponding label after segmentation. Note that the defective points are labeled “1”.

#### 3.2.5. Result Synthesization

Result synthesization is conducted mainly to improve the robustness and accuracy of the defect inspection task. Here, we follow the strategy given in [35,36], in which the authors combine information from multiple pyramid levels. This method assumes that a defect must appear in at least two adjacent levels. Therefore, a logical AND operation can be implemented in every pair of adjacent levels to reduce false detections. This operation is expressed as:

(10) $$\varsigma^{\left(i,i+1\right)}\left(x\right)=\varsigma^{\left(i\right)}\left(x\right)\;\&\;\varsigma^{\left(i+1\right)}\left(x\right).$$

Note that each residual map is scaled up to be the same size as the original input images. The symbol “&” refers to the AND operation between pixels at the same position in two adjacent maps. Next, the resulting maps are associated with a logical OR operation to generate the final result. That is:

(11) $$\varsigma \left(x\right)=\varsigma^{\left(1,2\right)}\left(x\right)\;\left|\;\varsigma^{\left(2,3\right)}\left(x\right)\cdots \;\right|\;\varsigma^{\left(n-1,n\right)}\left(x\right),$$

where “|” refers to the OR operation between pixels at the same position, and $\varsigma \left(x\right)$ is considered the final consolidated map. Note that a morphologically open operation can be carried out to remove noise interference if necessary.

## 4. Experiments and Discussion

In this section, we will first introduce the datasets and evaluation criteria used in the subsequent experiments. Then, several sets of experiments are presented to evaluate the performance of the proposed MSCDAE model. Detailed descriptions are given below.

### 4.1. Datasets and Evaluation Criteria

**Figure 7:**
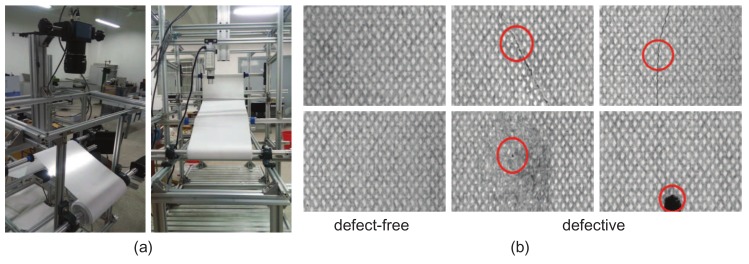
(**a**) The automatic optical inspection test bench utilized for fabric defect detection; (**b**) Defect-free and defective samples collected by the test bench.

Four datasets were utilized in this study: Fabrics [37], KTH-TIPS [38], Kylberg Texture [39], and ms-Texture, which we created. The Fabrics dataset consists of approximately 2000 samples of garments and fabrics, with some of them captured under different illumination conditions. The KTH-TIPS image database was created to extend the CURet database in two directions, providing variations in scale as well as pose and illumination, by imaging other samples of a subset of its materials in different settings [38]. It is an challenging dataset for defect detection. Kylberg Texture is also a widely used database of 28 texture classes. Patterned textures in this database are very different from each other. All patches have been normalized for convenient processing. The ms-Texture is a dataset created with our automatic optical inspection (AOI) test bench, as shown in Figure 7a. Samples are collected with a line-scan CCD camera and may have multiple different types of defects. Some typical defect-free and defective samples are shown in Figure 7b. In later experiments, our models are verified with these databases to evaluate their robustness and stability.

**Figure 8:**
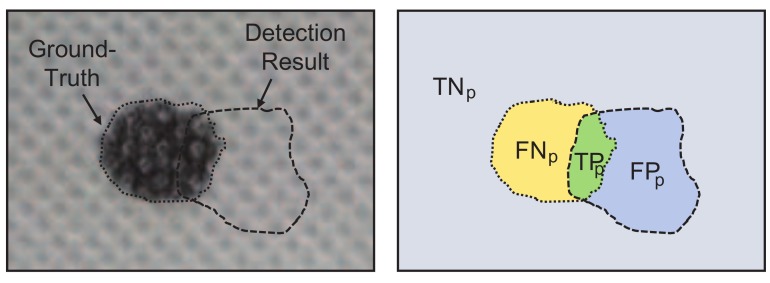
Definition of the ${TN}_{p}$, ${FN}_{p}$, ${TP}_{p}$, and ${FP}_{p}$ indicators.

The evaluation criteria utilized in our experiments include two aspects: image-level and pixel-level performance metrics. The former measure the accuracy of tagging images as defective or defect-free. They are widely used metrics for defect inspection and include the detection rate $D_{R}$, false alarm rate $F_{R}$, and detection accuracy $D_{Acc}$. These metrics are defined as follows:

(12) $$\begin{array}{cc} D_{R} & =\frac{TP}{N_{\mathrm{defect}}}\times 100\%,\\ F_{R} & =\frac{FP}{N_{\mathrm{defect}-\mathrm{free}}}\times 100\%,\\ D_{Acc} & =\frac{TP+TN}{TP+FN+TN+FP}\times 100\%, \end{array}$$

where *TP* and *FN* refer to the ratios of defective samples that are detected as defective and defect-free, respectively. *TN* and *FP* refer to the ratios of defect-free samples that are correctly detected as defect-free and falsely detected as defective, respectively. Analogously, $N_{\mathrm{defect}-\mathrm{free}}$ and $N_{\mathrm{defect}}$ designate the total numbers of corresponding samples. Pixel-level metrics evaluate the inspection accuracy by directly measuring the predicted pixels. As shown in Figure 8, ${TP}_{p}$ refers to the proportion of correctly segmented defective areas in the foreground, and ${FP}_{p}$ refers to the proportion of falsely segmented defective areas in the background. The meanings of indicators ${TN}_{p}$ and ${FN}_{p}$ are similar. Therefore, the quantitative inspection performance can be evaluated with:

(13) $$\begin{array}{cc} Recall & =\frac{{TP}_{p}}{{TP}_{p}+{FN}_{p}}\times 100\%,\\ Precision & =\frac{{TP}_{p}}{{TP}_{p}+{FP}_{p}}\times 100\%,\\ F1-Measure & =\frac{2\cdot Precision\cdot Recall}{Precision+Recall}\times 100\%, \end{array}$$

where the *F1-Measure* indicator is a comprehensive evaluator that uses both the Recall and Precision indicators.

### 4.2. Analysis of the Defect Detection Principle

As stated before, the proposed MSCDAE model is a defect detection architecture based on a Gaussian pyramid, and the final detection result of this model is the synthesis of results from multiple pyramid scales. Whether this fusion mechanism is beneficial to the overall performance still needs to be validated.

**Figure 9:**
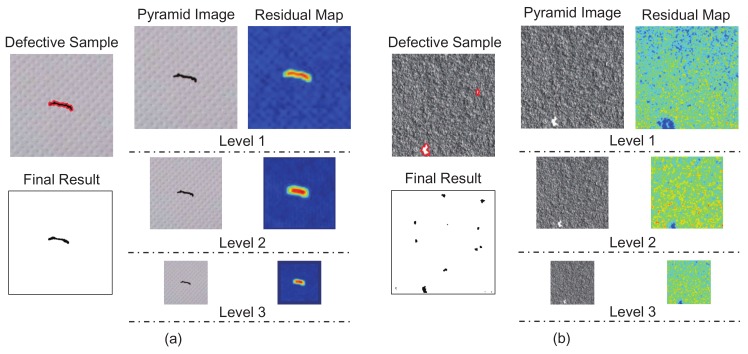
Final and intermediate defect detection results of the proposed MSCDAE model on (**a**) cotton and (**b**) canvas fabrics.

Figure 9 shows the detection results for two typical fabric samples. Both the final and intermediate results given by the proposed MSCDAE model are shown. Note that when synthesizing the final results, the reconstruction residual map in each pyramid layer is interpolated to the same size as the defective raw image sample. By analyzing these results, we can conclude the following: (1) The sensitivity of the trained CDAE network in different pyramid layers is different according to the texture information, so the detection results of the defects are also different. As shown in Figure 9a, the repetitive textures have nearly disappeared in level 3. Therefore, defects tend to be inspected easier in this level. However, compared with the results in level 1, the results from high resolution scales are relatively coarse. Thus, synthesizing results from different levels is more favorable for obtaining more accurate results; (2) The utilized fusion mechanism is capable of enhancing robustness and eliminating false detections. As shown in Figure 9b, the small defect at the top right of the figure has vanished in level 3. However, it can be accurately detected in level 1 and level 2. Many small defects are over-detected in level 1. However, they can be corrected in level 2 and level 3. Therefore, the proposed MSCDAE model tends to be more robust and accurate for defect inspection than models with only a single resolution scale.

### 4.3. Evaluation of Defect Detection Performance

**Figure 10:**
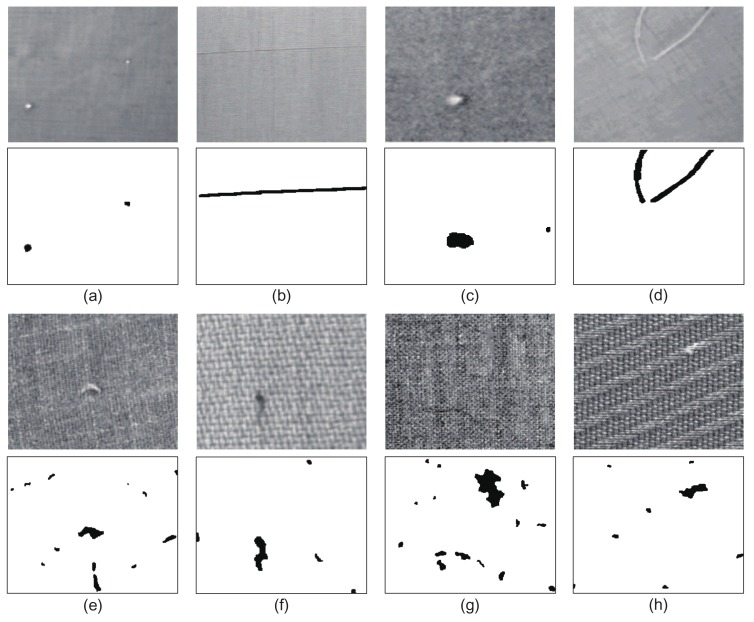
Defect inspection results using the proposed MSCDAE model. The images (**a**)–(**d**) and (**e**)–(**h**) belong to the p1 and non-p1 types of defects, and they are representative samples in each sample series.

As stated in Section 4.2, the Gaussian pyramid architecture and reconstruction residual mechanism utilized in the proposed MSCDAE model are capable of improving the robustness and accuracy for fabric defect detection. Here, we will verify this statement. In Figure 10, we show the results obtained for several typical fabric defects using the MSCDAE model. Each fabric sample comes from different textural series with 20 samples. These results are the final prediction performance after synthesis. As a whole, the MSCDAE model shows good performance for both *p*1 and non-*p*1 defects. In addition, it has good adaptability for defects of different scales, e.g., the defects in Figure 10a,d. Moreover, it can be seen from this figure that for samples with relatively complex patterned textures, defective regions tend to be over-detected with the proposed model. This phenomenon is mainly due to the fact that the texture characteristics of these texture types are not completely repetitive but have anisotropy in local regions, e.g., the samples in Figure 10f–h.

Table 1 shows average quantitative results for the proposed model on these defective sample series, and similar conclusions can be drawn from these data. Specifically, the MSCDAE model shows good performance on the Recall indicator, which is utilized to evaluate the detection accuracy. The results for the Precision indicator indicate that the proposed model has good suppression ability for over-inspection and missing inspection, to a certain extent. Furthermore, as shown in Table 2, the detection performance on the comparison datasets using the image-level criteria is verified. It can be seen that the overall inspection accuracy reaches over 80.0% on all these datasets. This experimental result further demonstrates the robustness of the MSCDAE model.

### 4.4. Comparison of Defect Detection Performances

To verify the performance of the proposed MSCDAE model more fully, in this section, we will compare the inspection results of this model with those of several unsupervised algorithms, specifically the discrete cosine transform (DCT) [40], the phase only transform (PHOT) [10] and the nonlocal sparse representation (NLSR) [41] approaches. The DCT model is a typical defect inspection method that tries to eliminate random texture patterns and retain anomalies in the restored image by reconstructing the frequency matrix without selected large-magnitude frequency values. The PHOT approach is based on the discrete Fourier transform and attempts to remove any regularities and preserve only irregular patterns for defect inspection. The NLSR is an efficient defect detection model based on a non-locally centralized sparse representation. Nonlocal similarities in the fabric structure information are exploited to improve the image restoration effect, thereby increasing the defect detection rates.

**Figure 11:**
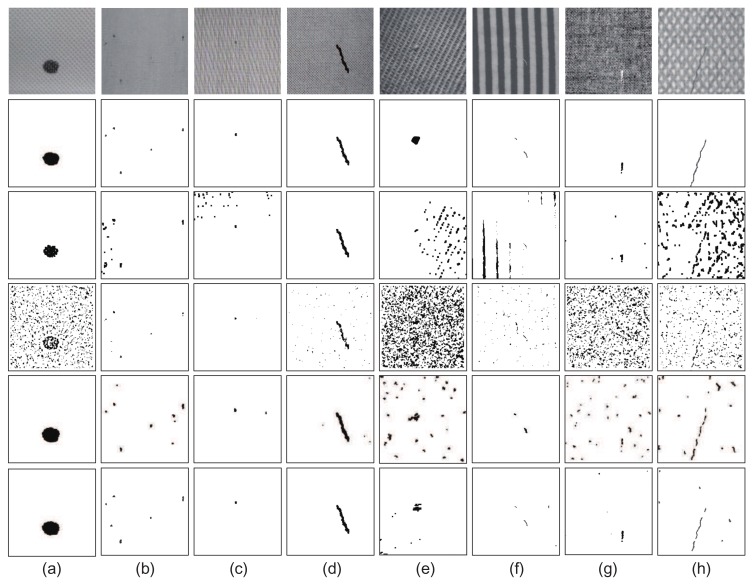
Defect detection results obtained with different methods for various textural samples. Top to bottom are the original defective samples, ground-truth regions, and results obtained by the DCT, PHOT, NLSR and MSCDAE models. Defects (**a**) to (**h**) are representative samples in each sample series.

As shown in Figure 11, we compared the performance of these methods on several patterned textures of *p*1 and non-*p*1 types. According to these experimental results, it can be seen that the DCT-based method exhibits good performance for conventional fabric textures, e.g., the samples in Figure 11a,d,g. However, for relatively more complex fabric textures, e.g., the samples in Figure 11e,f,h, repetitive textures cannot be absolutely removed in the restored images because of differences in local texture characteristics and defect scales. These factors may affect the accuracy of the defect detection process. The PHOT approach is also a method that presents good performance, especially for defects with small sizes, e.g., the samples in Figure 11b,c. However, this method is greatly influenced by the segmentation threshold, and it fails for some textures of non-*p*1 types, such as the samples in Figure 11e,g. The NLSR is a method based on sparse coding and shows relatively similar performance to the proposed MSCDAE model. This method has certain adaptability to different types of fabric textures, but it can also be easily influenced by atoms in the learned dictionary because of the dictionary encoding mechanism utilized. In other words, the performance can be easily affected by the training samples. In addition, noise in the testing samples may also influence the final inspection results. In the proposed MSCDAE model, the effects of certain types of noise have been taken into consideration in the training process of the CDAE networks at each pyramid level, and thus, it tends to have better robustness to noise, e.g., the samples in Figure 11g,h.

Furthermore, as shown in Table 3, we quantified the average results of the compared methods on these sample series (pixel-level). According to these quantitative results, the following conclusions can be drawn: (1) On the whole, methods based on local image patches (NLSR and MSCDAE) tend to have better stability than those based on global image reconstruction (DCT and PHOT). The latter have relatively poor performance on samples in Figure 11e,f according to the *Recall* and *Precision* indicators; (2) The proposed MSCDAE model is a method with a multi-pyramid architecture, and the CDAE network at each scale is sensitive to textures with different scales. Therefore, it tends to have better robustness compared with the NLSR method; (3) The proposed MSCDAE model has the best comprehensive performance compared with all these methods. The pixel-level quantitative experimental results in this table also demonstrate the superiority and stability of the MSCDAE model.

Finally, we quantified the average image-level performance of the compared methods on the four datasets, as shown in Table 4. Similar conclusions can be drawn that methods based on local image patches (NLSR and MSCDAE) tend to have better detection accuracy than those based on global image reconstruction (DCT and PHOT) on the whole. The proposed MSCDAE mode have the best comprehensive performance compared with all the compared methods. This experiment further demonstrates the superiority of the MSCDAE model.

## 5. Implementation Details

The experiments in this manuscript are conducted on a server with 12 cores, 128 GB memory and GTX 980Ti Nvidia GPU. The languages utilized for the proposed MSCDAE model and the compared methods are Python and Matlab. Thanks to the deep learning library Keras [42] and the third-party libraries included in this module.

## 6. Conclusions

In this paper, an unsupervised and efficient fabric defect detection model, MSCDAE, based on multi-scale convolutional denoising autoencoder networks has been proposed. This model has the capacity to synthesize results from multiple pyramid levels, highlighting defective regions through the reconstruction residual maps generated with the CDAE networks. It is a generalized that which can be trained with only a small number of defect-free samples (no labeled ground truth or human intervention is needed). In addition, it has the ability to address multiple types of textile fabrics and defects. Visual and quantitative results on samples from the Fabrics, KTC-TIPS, Kylberg Texture and ms-Texture datasets have demonstrated the superiority and robustness of this model. In the future, more experiments will be carried out to further improve the accuracy and stability of this model for more complicated patterned fabric textures.

## Figures and Tables

**Table 1:** Quantitative pixel-level defect detection results for the proposed MSCDAE model on samples from (a) to (h) series (each series contains 20 images with real defects).

Criterion(%)	(a) Series	(b) Series	(c) Series	(d) Series	(e) Series	(f) Series	(g) Series	(h) Series
*Recall*	0.5316	0.6102	0.9177	0.8366	0.9098	0.7936	0.9521	0.9355
*Precision*	0.6531	0.7349	0.6453	0.7573	0.4251	0.6517	0.3758	0.3942
*F1-Measure*	0.5861	0.6667	0.7578	0.7950	0.5794	0.7157	0.5389	0.5547

**Table 2:** Quantitative image-level defect detection results for the proposed MSCDAE model on the Fabrics, KTH-TIPS, Kylberg Texture and ms-Texture datasets.

Criterion(%)	$D_{R}$	$F_{R}$	$D_{\mathrm{Acc}}$
Fabrics (62 samples)	87.5 (21/24)	18.4 (7/38)	83.8
KTH-TIPS (128 samples)	84.1 (37/44)	14.3 (12/84)	85.2
Kylberg Texture (132 samples)	85.3 (29/34)	21.4 (21/98)	80.3
ms-Texture (50 samples)	84.6 (11/13)	16.2 (6/37)	84.0

**Table 3:** Comparison of quantitative pixel-level segmentation results obtained with different methods for various textural sample series (each series contains 20 samples with real or synthetic defects).

	Criteria	*Recall*				*Precision*				*F1-Measure*			
Samples		DCT	PHOT	NLSR	Ours	DCT	PHOT	NLSR	Ours	DCT	PHOT	NLSR	Ours
(a) series		0.7352	0.6131	**0.9325**	0.9256	**0.8985**	0.2417	0.7148	0.8353	0.8087	0.0.3467	0.8093	**0.8781**
(b) series		0.7912	0.5484	0.7743	**0.7959**	0.3251	**0.7254**	0.4487	0.6584	0.4608	0.6246	0.5682	**0.7206**
(c) series		**0.8359**	0.5495	0.7018	0.7347	0.1025	0.8571	0.4497	**0.8953**	0.1826	0.6697	0.5482	**0.8071**
(d) series		0.8547	0.6988	0.8416	**0.9451**	**0.8222**	0.6134	0.5491	0.8121	0.8381	0.6533	0.6646	**0.8736**
(e) series		0.3035	0.4862	**0.5351**	0.4795	0.1032	0.2142	0.1749	**0.6024**	0.1540	0.2974	0.2636	**0.5540**
(f) series		0.2435	0.5101	0.7482	**0.8353**	0.1759	0.1016	0.5412	**0.6333**	0.2043	0.1694	0.6281	**0.7204**
(g) series		**0.8912**	0.1117	0.6381	0.6479	0.3540	0.1984	0.2264	**0.3951**	**0.5067**	0.1429	0.3342	0.4909
(h) series		**0.7781**	0.5426	0.4105	0.7414	0.1684	0.1158	0.3003	**0.6357**	0.2769	0.1909	0.3469	**0.6845**

**Table 4:** Comparison of quantitative image-level defect detection results for the compared algorithms on the Fabrics, KTH-TIPS, Kylberg Texture and ms-Texture datasets.

Accuracy (%)	DCT	PHOT	NLSR	Ours
Fabrics (62 samples)	71.0	62.9	79.0	**83.8**
KTH-TIPS (128 samples)	69.5	64.8	75.8	**85.2**
Kylberg Texture (132 samples)	76.5	68.2	**81.1**	80.3
ms-Texture (50 samples)	78	54.0	68.0	**84.0**

## Abbreviations

The following abbreviations are used in this manuscript:

AE	Autoencoder
AOI	Automatic optical inspection
CAE	Convolutional autoencoder
CCD	Charge coupled device
CDAE	Convolutional denoising autoencoder
DCT	Discrete cosine transform
GPU	Graphics processing unit
MSCDAE	Multi-scale convolutional denoising autoencoder
NLSR	Nonlocal sparse representation
PCA	Principal component analysis
PHOT	Phase only transform
WLD	Weber local descriptor
